# Interplay of diverse adjuvants and nanoparticle presentation of native-like HIV-1 envelope trimers

**DOI:** 10.1038/s41541-021-00364-x

**Published:** 2021-08-17

**Authors:** Kwinten Sliepen, Edith Schermer, Ilja Bontjer, Judith A. Burger, Réka Felfödiné Lévai, Philipp Mundsperger, Philip J. M. Brouwer, Monica Tolazzi, Attila Farsang, Dietmar Katinger, John P. Moore, Gabriella Scarlatti, Robin J. Shattock, Quentin J. Sattentau, Rogier W. Sanders

**Affiliations:** 1 Department of Medical Microbiology, Amsterdam Institute for Infection and Immunity, Amsterdam UMC, University of Amsterdam, Amsterdam, The Netherlands; 2 Control Laboratory of Veterinary Medicinal Products and Animal Facility, Directorate of Veterinary Medicinal Products, National Food Chain Safety Office, Budapest, Hungary; 3 Polymun Scientific Immunbiologische Forschung GmbH, Klosterneuburg, Austria; 4 Viral Evolution and Transmission Unit, Division of Immunology, Transplantation and Infectious Diseases, IRCCS Ospedale San Raffaele, Milan, Italy; 5 Department of Microbiology and Immunology, Weill Medical College of Cornell University, New York, NY USA; 6 Imperial College London, Department of Medicine, Division of Infectious Diseases, Section of Virology, Norfolk Place, London, W21PG UK; 7 The Sir William Dunn School of Pathology, The University of Oxford, Oxford, OX13RE UK

**Keywords:** Adjuvants, Recombinant vaccine, Protein vaccines, HIV infections

## Abstract

The immunogenicity of HIV-1 envelope (Env) trimers is generally poor. We used the clinically relevant ConM SOSIP trimer to compare the ability of different adjuvants (squalene emulsion, ISCOMATRIX, GLA-LSQ, and MPLA liposomes) to support neutralizing antibody (NAb) responses in rabbits. The trimers were administered as free proteins or on nanoparticles. The rank order for the adjuvants was ISCOMATRIX > SE > GLA-LSQ ~ MPLA liposomes > no adjuvant. Stronger NAb responses were elicited when the ConM SOSIP trimers were presented on ferritin nanoparticles. We also found that the GLA-LSQ adjuvant induced an unexpectedly strong antibody response to the ferritin core of the nanoparticles. This “off-target” effect may have compromised its ability to induce the more desired antitrimer antibodies. In summary, both adjuvants and nanoparticle display can improve the magnitude of the antibody response to SOSIP trimers but the best combination of trimer presentation and adjuvant can only be identified experimentally.

## Introduction

The need for an HIV-1 vaccine is undebated but formidable scientific challenges have hampered the development of a vaccine. Neutralizing antibodies (NAb) responses correlate with protection for many licensed antiviral vaccines^1^ but HIV-1 NAbs have been difficult to induce by vaccination. Given the huge viral sequence diversity, an HIV-1 vaccine would have to induce broadly neutralizing antibodies (bNAbs), i.e., NAbs that can cope with global, or at a minimum regional, HIV-1 diversity^2^. HIV-1 NAbs and bNAbs do develop during natural HIV-1 infection providing evidence that the human immune system can generate such antibodies^3^.

The induction of NAb responses against relatively neutralization-resistant (Tier 2) viruses by vaccination was facilitated by the design of stable soluble mimics of the native Env trimer, such as BG505 SOSIP.664^4^. Structure-based design led to newer generations of recombinant native-like trimers as well as trimers from circulating strains^5–8^. Immunogens based on consensus sequences might also be valuable in vaccine strategies aimed at inducing bNAbs, since consensus sequences are usually closer to circulating isolates than circulating isolates are to one another. Moreover, rare isolate-specific antigenic determinants are eliminated, favoring, at least in theory, more cross-reactive responses^9,10^. An example is the ConM SOSIP.v7 trimer that is based on a consensus sequence of all group M virus isolates. ConM SOSIP.v7 induces strong NAb responses against artificial consensus-based viruses. These NAb responses target the trimer apex, an epitope that might be an appropriate vaccine component to drive neutralization breadth^11^. Therefore, ConM SOSIP.v7 is now being evaluated in three human clinical trials (clinical trial.gov: NCT03961438, NCT03816137, NCT04046978).

While important steps have been made in generating immunogens that mimic the native Env trimer and induce NAbs, these immunogens do not address the relatively poor overall magnitude and durability of anti-Env responses, properties that appear to be independent from the antigenic conformation of Env immunogens^12^. Env is a notoriously poor immunogen in comparison with other pathogen-based immunogens^13^. Induction of a robust autologous Tier 2 NAb response usually requires at least three recombinant Env protein immunizations in rabbits or macaques^4,14–16^. In contrast, vaccination with recombinant influenza hemagglutinin glycoproteins or respiratory syncytial virus glycoprotein F elicits potent neutralizing responses after only one or two immunizations^17,18^.

The durability of Env-induced humoral responses is also poor. While the half-life of immunity induced by licensed protein subunit vaccines, such as those against tetanus and diphtheria, is 10–20 years, the half-life of anti-Env Ab responses induced by Env subunit vaccines is typically 30–60 days, i.e., >100-fold shorter^4,19,20^. Env has also been inferior as an immunogen in direct comparisons with influenza HA, rabies protein G and hepatitis B virus surface Ag^21–24^. The reasons are poorly understood but might relate in part to the immunosuppressive effect Env has on immune cells, involving interactions of mannose glycans with C-type lectin receptors^25–27^.

Adjuvants are capable of stimulating different arms of the immune system^28^ and are vital components of subunit vaccines, especially in the case of poorly immunogenic Env. However, some adjuvants are known to have adverse effects on antigen integrity. For example, some immunization studies suggest that Freund’s adjuvant can denature antigens and open up cryptic epitopes that are irrelevant for inducing NAbs^29,30^. A recent study revealed that the most commonly used adjuvants have little effect on SOSIP trimer integrity or epitope presentation^31^. However, adjuvants based on acidified alum and polyanionic CpG oligodeoxynucleotide decrease trimer stability and can block epitopes on the trimer apex, respectively.

Rabbits are commonly used in HIV-1 vaccine research because of their ability to induce NAbs against Tier 2 viruses in contrast to mice, possibly because the distribution of CDR lengths of rabbit immunoglobulins allow them to engage Env despite its extensive glycan shield^32^. Furthermore, the relatively large amounts of sera that can be obtained from rabbits allow for testing of a comprehensive number of variables. Importantly, the Env epitopes that are recognized by NAbs from rabbits are also targeted by NAbs that are induced in nonhuman primates and thus are very relevant for studying vaccine-induced NAbs in early preclinical settings^11,33–35^. Therefore, we aimed to compare different adjuvants on the induction of HIV-1 NAbs in rabbits.

We selected four adjuvants that are used in human studies and have no or only minimal effect on Env trimer structure^31^. ISCOMATRIX is a mix of QS21 saponin, cholesterol, and phospholipid and has been tested for safety in humans and has been used widely for rabbit immunization studies^4,32,36–39^. It is very similar to Matrix-M, the adjuvant component of Novavax’ SARS-CoV-2 vaccine^40^. Second, we tested a squalene oil-in-water emulsion (SE) that is comparable to the MF59^TM^ adjuvant that is used in human influenza vaccines^41^. Third, we evaluated a formulation of the TLR4 ligand glucopyranosyl lipid adjuvant with liposomal QS21 (GLA-LSQ)^42^. GLA-LSQ is used with BG505 SOSIP.664 trimers in a clinical study (NCT04177355) and is similar to the AS01B adjuvant that is used in a different trial with BG505 SOSIP.664 (NCT03699241) and a trial with the germline-targeting BG505 GT1.1 trimer (NCT04224701), as well as the RTS,S malaria vaccine^43^. We also tested monophosphoryl lipid A (MPLA), another TLR4 ligand, combined with liposomes^44^. The latter adjuvant is used in clinical trials with ConM SOSIP.v7 (NCT03816137, NCT03961438).

Another established strategy to improve humoral responses is by displaying antigens on nanoparticles. Nanoparticle presentation enhances the immunogenicity of antigens by increasing their avidity and thereby improving B cell receptor cross-linking and epitope avidity. Furthermore, nanoparticle presentation improves lymph node trafficking^45,46^. The notion that the only licensed viral protein subunit vaccines, those against hepatitis B, hepatitis E, and human papillomavirus, are virus-like particles illustrates that multimerized display of antigens is important for immunogenicity^45^. A number of nanoparticle platforms have been used to increase the immunogenicity of soluble Env trimers^47^. These platforms include the ferritin 24-mer that can present eight Env trimers^38,48–50^, liposomes with (non-) covalently linked Env trimers^51–54^, virus-like particles^55^, and computationally designed two-component nanoparticles^56,57^.

In this study, we systematically compared the immunogenicity of ConM SOSIP.v7 native-like trimers formulated in four different adjuvants or in the absence of adjuvant. In addition, we evaluated ConM SOSIP.v7 trimers presented on ferritin nanoparticles formulated in three different adjuvants. As expected, we found that adjuvants enhanced binding titers and the potency of the polyclonal NAb response, in particular early in the immunization regimen. Furthermore, nanoparticle presentation increased binding titers and the potency of the polyclonal NAb response, also particularly early on. Surprisingly, we found that adjuvants have different effects on the relative immunodominance/subdominance of the nanoparticle core and Env. This is relevant because we observed an inverse correlation between the relative strength of the Ab response against the nanoparticle core and the NAb response against HIV-1. Together, these results might be useful for selecting the optimal adjuvant for the induction of NAbs in particular when combining with nanoparticle display of Env trimers.

## Results

### Study design

**Fig. 1:**
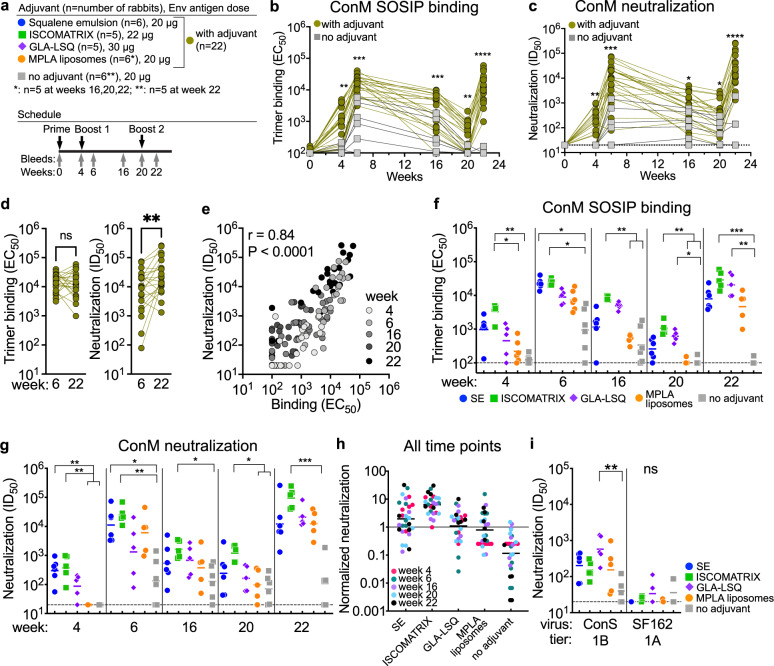
Effect of adjuvants on trimer immunogenicity in rabbits. **a** Schematic representation of the rabbit immunization schedule. Rabbits were immunized at weeks 0, 4, and 20 with ConM SOSIP.v7 trimer in the indicated adjuvants. Bleeds were taken at 0, 4, 6, 16, 20, and 22. Every color represents a specific adjuvant. The same color icons are used throughout all figures. **b** Midpoint serum-binding titers (EC_50_) measured against ConM SOSIP.v7 trimer in ELISA. Differences between nonadjuvanted and the pooled adjuvanted groups were compared at each time point by the Mann–Whitney *U* test. **c** Midpoint serum-neutralization titers (ID_50_) measured against ConM virus. Differences between nonadjuvanted and the pooled adjuvanted groups were compared at each time point by the Mann–Whitney *U* test. **d** Comparison of binding and neutralization titers two weeks after the second (week 6) and third immunization (week 22) from the pooled adjuvanted trimer group. A Wilcoxon test was used to determine differences. **e** Simple linear regression analysis of the midpoint binding titers and ConM neutralization titers over all postprime time points. The Spearman *r* values and p-values are indicated. **f** Comparison of the midpoint trimer binding titers between the adjuvanted trimer groups. **g** Comparison of the ConM neutralization titers between the different adjuvants. **h** Each individual ID_50_ titer was normalized against the corresponding geometric mean ID_50_ normalized to 1.0 (horizontal line). Shown are the pooled normalized ConM neutralization titers from all postprime time points. **i** Week 22 serum-neutralization titers against Tier 1B ConS virus and Tier 1 A SF162. Stars denote statistical differences: *p < 0.05; **p < 0.01; ***p < 0.001 determined by Kruskal–Wallis test, followed by Dunn’s post-test unless otherwise noted. (**h**) contains published data, see also Supplementary Table 2^11,56^. All data were generated at the AMC. Horizontal lines represent geometric mean values.

**Fig. 2:**
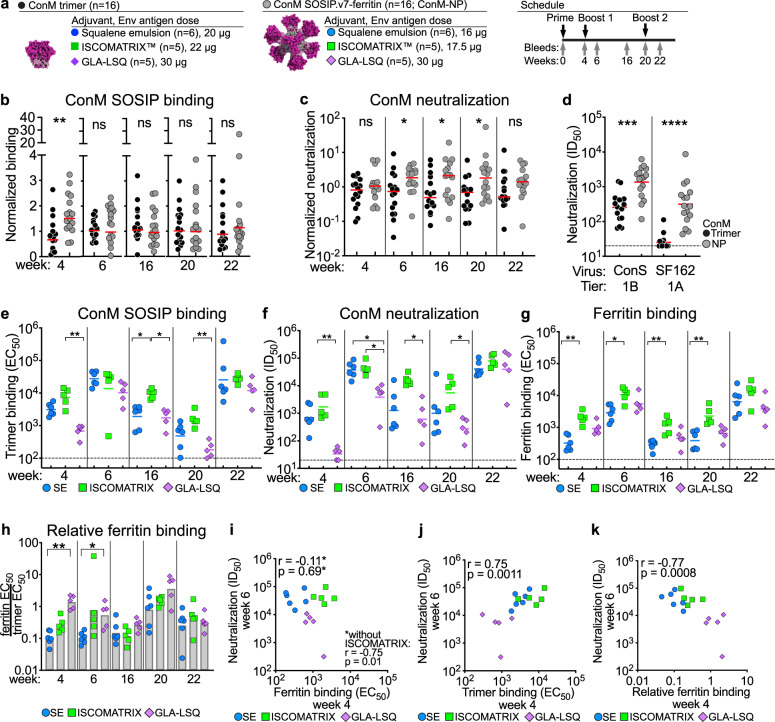
Immunogenicity of adjuvanted ConM SOSIP.v7-ferritin in rabbits. **a** Rabbit immunization groups and schedule. The three ConM SOSIP.v7-ferritin nanoparticle (ConM-NP) groups with ISCOMATRIX, SE, and GLA-LSQ are compared to trimers in the same three adjuvants. **b** ConM SOSIP.v7 binding titer comparison from ELISA. Serum-binding EC_50_ titers of rabbits were normalized for each adjuvant to allow a comparison between the trimer and ferritin group. The normalized EC_50_ titers of the sixteen ConM trimer or ConM-NP immunized rabbits were compared at every time point. **c** Neutralization titers against ConM virus by nanoparticle or trimer immunized rabbits. The neutralization titers (ID_50_) of rabbits were first normalized for each adjuvant to allow a comparison between the trimer and nanoparticle group. **d** Neutralization titers (ID_50_) from week 22 against ConS or SF162 virus induced by trimers or nanoparticles in rabbits. Data were published before in^11,56^, see also Supplementary Table 2. **e** Binding titer comparison of the different adjuvanted ConM-NP groups. **f** Neutralization titers of sera from rabbits that received ConM-NPs with either SE, ISCOMATRIX, or GLA-LSQ. **g** Midpoint binding titers against the ferritin cage by ConM-NP immunized rabbits. Data are represented as geomeans + geomean SD from five or six biological replicates. Stars denote significant differences between the ISCOMATRIX and SE groups by Kruskal–Wallis test, Dunn’s post-test. **h** Relative ferritin binding by ConM-NP immunized rabbits. Ratios are derived from the midpoint binding titers of Figs. 2E and 2G. **i** Simple linear regression analysis of ferritin binding at week 4 (**g**) and ConM neutralization at week 6 (**f**). **j** Simple linear regression analysis of SOSIP binding at week 4 (**e**) and ConM neutralization at week 6 (**f**). **﻿k** Simple linear regression analysis of relative ferritin binding at week 4 (**h**) and ConM neutralization atweek 6 (**﻿f**). Spearman *r* and *p* value are indicated. Stars denote statistical differences: *p < 0.05; **p < 0.01; ***p < 0.001. See also Supplementary Table 2. All data were generated at the AMC. Horizontal lines represent geometric mean values.

The purpose of our study was to determine the effect of different adjuvants and ferritin nanoparticle presentation on the immunogenicity of soluble Env trimers. We chose ConM SOSIP.v7 as a model antigen because it is relatively immunogenic compared to other soluble Env trimers, which allows us to detect potential differences between groups, including those that might arise early in the immunization schedule. The experimental groups are derived from three different rabbit immunization studies, including two of which we have reported on previously^11,56^. In each study, the rabbits were immunized at the same intervals (weeks 0, 4, and 20) as used previously with other SOSIP trimers^4,7,8^. The rabbits were culled at week 22 when antibody levels are expected to peak. The immunogen Env doses differed less than twofold between all groups (16–30 µg Env content per dose, Fig. 1a and Fig. 2a) across the three experiments (see Materials and methods for specific details). Here, we have analyzed these sera in binding ELISAs and ConM virus neutralization assays to assess the impact of adjuvants and ferritin nanoparticle presentation on Env immunogenicity (Figs. 1a and 2a).

In the first immunization study, six rabbits per group were immunized with 20 µg of soluble ConM SOSIP.v7 trimers (ConM trimers) with squalene emulsion (SE), MPLA liposomes, or without adjuvant. Another group received 20 µg ConM SOSIP.v7-ferritin nanoparticles (ConM-NPs) formulated in SE (16 µg Env equivalent). In the second study, five rabbits per group received 22 µg soluble ConM trimers or ConM-NPs (17 µg Env equivalent) formulated in 75 units ISCOMATRIX adjuvant^11^. In the third study, five rabbits per group received 30 µg of soluble ConM trimers or 38 µg ConM-NPs (30 µg Env equivalent) formulated in 25 µg GLA-LSQ^56^. The adjuvanted ConM trimer groups are summarized in Fig. 1A and the three ConM-NP groups are summarized in Fig. 2A.

### Adjuvants increase binding and neutralizing Ab responses in rabbits

First, we compared the humoral responses induced by the adjuvanted group of ConM trimers (*n* = 22 in total) to the nonadjuvanted trimers (*n* = 6). As expected, the adjuvanted trimer immunized rabbits induced higher binding Ab responses than rabbits immunized without adjuvants (from ~6 up to ~100-fold higher depending on the time point) (Fig. 1b). We already detected midpoint binding Ab titers (EC_50_) above 100 in most sera from adjuvanted ConM SOSIP.v7 trimer immunized rabbits after the first immunization. In contrast, to detect similar EC_50_ binding titers most other adjuvanted SOSIP trimer immunogens require at least two doses^14,16^. After the second immunization, the geometric mean titer of the combined adjuvanted trimer group was ~14-fold higher than the nonadjuvanted group. As with other Env immunizations, the binding responses waned over time and were boosted by the last immunization. We did not observe differences in decay rates between adjuvanted and nonadjuvanted trimers (Fig. 1b).

A number of rabbits elicited detectable ConM NAb responses after only one immunization (week 4, Fig. 1c). The combined adjuvant group induced significantly higher NAb responses throughout the immunization schedule than the nonadjuvanted trimer group (~sixfold, ~50-fold, and ~200-fold at weeks 4, 6, and 22, respectively) (Fig. 1c). Interestingly, binding titers did not increase with a third immunization (*p* = 0.63, Wilcoxon test), while neutralization titers increased significantly (*p* = 0.0038, Wilcoxon test) (Fig. 1d), suggesting that the third immunization triggered additional Ab maturation.

As expected, the titers in the neutralization assays correlated strongly with titers from the binding ELISA at each time point (Spearman *r* = 0.84, *p* < 0.0001 for all datapoints combined; *r* = 0.68–0.85, *p* ≤ 0.0001 for each time point) (Fig. 1e).

### Effect of different adjuvants on ConM SOSIP trimer immunogenicity

Next, we investigated the immunogenicity of the different trimer/adjuvant combinations. Notably, sera from rabbits immunized with MPLA liposomes displayed significantly lower binding Ab responses than ISCOMATRIX at weeks 4, 16, and 20 (*p* = 0.025, *p* = 0.0071, and *p* = 0.0038, respectively) (Fig. 1f and Supplementary Table 1). ISCOMATRIX adjuvanted trimers induced a trend toward the highest binding titers (not statistically significant) but after the third immunization the different adjuvant groups displayed no significant differences in binding titers (Fig. 1f).

The results from neutralization assays revealed a similar picture to the binding ELISAs. Most notably, none of the rabbits that received MPLA liposomes induced ConM NAbs 4 weeks after the prime, while 12/16 rabbits receiving trimers in SE, ISCOMATRIX or GLA-LSQ adjuvants displayed NAb ID_﻿50_ titers > 100 (Fig. 1g). At week 6, after the first boost, most rabbit sera contained strong neutralization activity with most ID_50_ titers > 1000 (Fig. 1g). As expected, these titers waned over time: 16 weeks after the first boost (week 20), NAb titers of rabbits that received ISCOMATRIX were still relatively strong (all five rabbits displayed ID_50_ titers > 500), while NAb titers from rabbits that received MPLA were significantly weaker (Fig. 1g). After the third immunization we did not detect significant differences between the adjuvants (Fig. 1g). To allow quantification of neutralization over the course of the experiment we normalized the individual ID_50_ titers to the geometric mean titer of the corresponding time point (week 4, 6, 16, 20, or 22). All normalized titers from the ISCOMATRIX group were above 1.0, which indicates that all ISCOMATRIX ID_50_ titers were higher than their respective geometric mean ID_50_ titers at each time point.

We tested the week 22 sera against the related, but more neutralization-resistant ConS virus and the sensitive heterologous Tier 1 A SF162 virus. ConS is based on a consensus sequence of group M, but it contains longer variable loops that are also different in sequence compared to ConM virus^11^. At week 22, virtually all trimer immunized rabbits, except some rabbits in the nonadjuvanted and MPLA liposome adjuvanted trimer group, had developed NAb activity (ID_50_ titer > 40) against ConS. None of the trimer groups showed strong neutralization against the SF162 virus or displayed neutralization breadth (Fig. 1h and Supplementary Table S2).

### Nanoparticle presentation enhances NAb responses

Rabbits immunized with ConM-NP received ISCOMATRIX, SE, or GLA-LSQ adjuvants (Fig. 2a). We compared the serum binding and neutralization titers from the rabbits that received ConM trimers with ConM-NPs by normalizing the titers for each adjuvant (Figs. 2b, 2c). At week 4, the normalized binding titers of the combined NP group (*n* = 16) were slightly higher (twofold) than those of the combined trimer group (*n* = 16) (*p* = 0.0044 for the comparison). After subsequent boosts, the combined normalized binding titers were similar between the ConM trimer and ConM-NP groups (Fig. 2b).

ConM neutralization was more strongly augmented by NP presentation than ConM binding (Fig. 2c). The combined ConM-NP group displayed significantly stronger NAb responses at weeks 6 (~threefold, *p* = 0.019), 16 (*p* = 0.035), and 20 (*p* = 0.012), and we observed a trend for week 22 (*p* = 0.051), interestingly we did not observe a significant difference at week 4. Over the course of the experiment, ConM neutralization was ~2.5-fold higher for NP immunized animals compared to trimer immunized animals (Fig. 2c, all weeks combined, *p* < 0.0001 for the comparison). As expected, binding Ab titers from ConM-NP immunized rabbits correlated strongly with NAb titers (Supplementary Fig. 1a).

Furthermore, rabbits immunized with nanoparticles also displayed significantly higher neutralization activity against ConS (geometric mean ID_50_ of 1368 for the NP group vs. 256 for the single trimer group, *p* = 0.0001) and SF162 (geometric mean ID_50_ of 315 for the NP group vs*.* 25 for the single trimer group, *p* < 0.0001) (Fig. 2d). The increased neutralization observed for SF162 probably represents anti-V3 Abs that are induced by the exposed V3-loop of uncleaved ConM SOSIP.v7 trimers that exist on ferritin nanoparticles^11^. We did not observe an improvement in neutralization breadth (Supplementary Table 2).

In summary, ferritin nanoparticle presentation significantly increased the immunogenicity of ConM SOSIP.v7 in rabbits.

### Adjuvants affect nanoparticle immunogenicity

Next, we compared the three adjuvants between the ferritin immunized animals. Overall, binding and neutralizing Ab titers were similar between SE and ISCOMATRIX nanoparticle immunized rabbits, except for the binding titers at week 16 (Figs. 2e, f). However, GLA-LSQ with ConM-NPs induced significantly lower binding Ab and NAb responses than ISCOMATRIX at most time points (Figs. 2e, f). Only two weeks after the third immunization (week 22), binding and neutralization titers became similar (Figs. 2e, f). Most notably, at week 4, none of the animals receiving nanoparticles with GLA-LSQ-induced NAb titers above 100, while SE and ISCOMATRIX nanoparticle immunized animals induced responses with ID_50_ values of 127–4860 (Fig. 2f). The geometric mean NAb titer of the GLA-LSQ group remained lower, even after the second immunization: ~10-fold lower at week 6 (*p* = 0.0198 and *p* = 0.0194, vs. SE and ISCOMATRIX, respectively), ~25-fold at week 16 (*p* = 0.029, *versus* ISCOMATRIX) and 20-fold at week 20 (*p* = 0.029, vs. ISCOMATRIX) (Fig. 2f). However, after the third immunization, NAb titers between the three groups were similar (Fig. 2f).

### Adjuvants impact anti-ferritin binding responses

The proteinaceous ferritin moiety of ConM-NPs is relatively exposed and the heterologous ferritin nanoparticle can induce an antiparticle Ab response^11,17^. B cells recognizing the ferritin moiety might compete with more useful B cells that recognize Env-specific epitopes. To investigate whether the different adjuvants impact the immunogenicity of the ferritin moiety we used an ELISA assay to measure binding Ab responses to the naked ferritin cage (Supplementary Table 3).

All ConM-NP immunized rabbits induced binding Ab responses to the ferritin moiety immediately after the first immunization and these responses were boosted after subsequent immunizations (Fig. 2g). The ISCOMATRIX recipients showed significantly greater reactivity to the ferritin moiety than SE at weeks 4, 6, 16, and 20 (Fig. 2g). When plotting the ratio of anti-ferritin titers to anti-Env titers to gauge the relative immunodominance of the two components, it is evident that GLA-LSQ causes a ~12-fold shift in immunodominance of ferritin compared to SE at week 4 (*p* = 0.0013) and ~fivefold at week 6 (*p* = 0.0205) (Fig. 2h). That difference was somewhat less pronounced and not statistically significant when comparing GLA-LSQ versus ISCOMATRIX.

We wished to assess the role of pre-existing anti-NP responses on anti-Env responses. Therefore, we plotted the week four ferritin ELISA binding titers (from Fig. 2g) vs. ConM neutralization at week 6 and we observed an inverse correlation trend, which was significant when we excluded the ISCOMATRIX group (Fig. 2i). As expected, week 4 trimer binding responses were predictive for week 6 neutralization (Fig. 2j). Furthermore, relative ferritin binding at week 4 (from Fig. 2h) was correlated with week 6 neutralization (*r* = −0.77; *p* = 0.0008, Fig. 2k). At later time points, we did not observe correlations between anti-ferritin responses and neutralization. These results suggest that early anti-ferritin responses induced by ConM-NPs might interfere with the development of ConM NAb responses when using certain adjuvants. In summary, adjuvants can alter the relative immunodominance of the ferritin cage vs. the ConM SOSIP.v7 trimer.

## Discussion

Here, we have evaluated the effect of adjuvants and nanoparticle presentation combined with a native-like HIV-1 Env trimer immunogen on the induction of Ab responses in rabbits.

As expected, adjuvants strongly augmented the binding and neutralizing Ab responses induced by ConM SOSIP.v7 trimers compared to when no adjuvant was used. Between the four trimer adjuvant groups, binding and neutralization titers did not significant differ after the last time point (week 22).

However, we found significant differences at earlier time points. Notably, after the first immunization, none of the rabbits that received MPLA liposomes induced a detectable ConM NAb response, while sera from most other rabbits did neutralize ConM efficiently after just one immunization. Overall, the adjuvant rank order for inducing NAbs using ConM SOSIP trimer in rabbits is as follows: ISCOMATRIX > SE > GLA-LSQ ≈ MPLA liposome > no adjuvant.

Nanoparticles were most immunogenic when combined with ISCOMATRIX, while they induced significantly lower anti-Env responses when combined with GLA-LSQ. Here, we have also shown that the ferritin moiety induces a rapid Ab response. This might lead to unwanted off-target anti-nanoparticle responses^13^. In contrast to its weak anti-Env response, GLA-LSQ caused a strong anti-nanoparticle response. This demonstrates that the strength of the anti-nanoparticle response depends on the adjuvant used. This might be explained by differences in antigen retention at the injection site and subsequent antigen presentation in germinal centers by adjuvant-recruited immune cells. Furthermore, SOSIP trimers combined with GLA-LSQ display a slightly decreased binding to the apex-targeting PGT145 bNAb^31^. This suggests that GLA-LSQ causes minor perturbations in the immunodominant apex of ConM SOSIP, which decreases induction of (N)Abs.

Whether or not anti-particle responses have adverse consequences for the quality of the NAb response was thus far unproven. Basic B cell immunology dictates that B cells compete in germinal centers to sequester antigen. In this case, B cells that recognize the surface of the nanoparticle compete for the same antigen as Env-specific B cells^58^. Thus, multivalent antigen display on proteinaceous nanoparticles acts as a double-edged sword: it increases immunogenicity of the antigen on display but it also introduces the nanoparticle moiety as a distraction. Previous studies have shown that pre-existing immunity to ferritin or other nanoparticles does not adversely affect Ab responses against RSV F and influenza HA antigens displayed on the same nanoparticles^17,59^. However, our results suggest that pre-existing anti-ferritin Ab responses might decrease HIV-1 Env NAb responses (Fig. 2i). This discrepancy might be rooted in the weaker immunogenicity of HIV-1 Env compared to influenza HA or RSV F^13^. Furthermore, the potential negative effects of pre-existing immunogenicity of the nanoparticle component might not have been detected in these studies with influenza HA and RSV F because of the limited numbers of animals in each group and the lack of diverse adjuvants.

More in-depth and focused studies will be needed to verify the role of nanoparticle responses in shaping the immunogenicity of the antigens displayed. Our study highlights that in cases when such responses are a problem, a carefully matched adjuvant might be part of the solution. Moreover, anti-nanoparticle responses can be reduced by adding glycans to mask nanoparticle epitopes^60^ or by using nanoparticles with less immunogenic surfaces, such as liposomes or host cell membrane-derived virus-like particles^52,55^.

The ferritin 24-mer we used here is a popular platform for the multivalent display of viral glycoproteins to increase their immunogenicity^11,17,38,50,61–63^. However, ferritin and many other nanoparticles fold intracellularly and this can lead to the occurrence of misfolded and non-native antigens on the surface of these nanoparticles. Indeed, SOSIP trimers on ferritin display increased binding to non-NAbs compared to their soluble counterparts^11^. Two-component nanoparticles, certain virus-like particles and liposomes enable the generation of nanoparticles that only display well-folded antigens because the antigens can be purified to high quality separately prior to mixing with the nanoparticle component^52,59,64^. The I53–50 nanoparticle is an excellent platform that enables the generation of two-component nanoparticles. Indeed, we recently showed that ConM SOSIP.v7 trimers on I53-50 nanoparticles induced significantly less undesired non-NAbs than ConM SOSIP.v7-ferritin probably because of the absence of uncleaved ConM SOSIP.v7 trimers that display non-NAb epitopes^11,56^.

In summary, our results inform the design of vaccination regimens aimed at inducing NAb responses. Our data imply that selecting the adjuvant of choice requires considering the nature of the antigen itself but also the higher order organization: soluble trimer or nanoparticle.

## Methods

### Proteins

The design and characterization of the ConM SOSIP.v7 and the ConM SOSIP.v7-ferritin proteins are described in detail previously^11^. Suspension 293 F were maintained in FreeStyle medium and transfected using 1 mg/mL PEImax at a density of 0.8–1.2 million cells/mL with a plasmid expressing ConM SOSIP.v7 or ConM SOSIP.v7-ferritin and a plasmid encoding furin. The supernatant was harvested 7 days after transfection, centrifuged, and filtered using Steritops (0.22 μm pore size; Millipore, Amsterdam, The Netherlands). Proteins were purified by adding the CNBr- activated sepharose 4B beads (GE Healthcare) carrying PGT145 to the filtered supernatant and incubated on a roller at 4 °C overnight. Subsequently, the supernatant and beads were passed over an Econo-Column chromatography column (Biorad). The column was then washed with three column volumes of 0.5 M NaCl and 20 mM Tris HCl pH 8.0. Protein was eluted with 3.0 M MgCl_2_ pH 7.5 and immediately buffer exchanged into TN75 buffer (75 mM NaCl, 20 mM Tris HCl pH 8.0) using a 100-kDa cut-off Vivaspin20 filter (Sartorius, Gӧttingen, Germany). The His-tagged ferritin nanoparticles were expressed in FreeStyle 293 F cells and purified by gravity flow over a Ni-NTA column (Qiagen) followed by SEC over a Superdex200 10/300 GL increase column. Fractions corresponding to the size of the ferritin 24-mer were pooled and concentrated in phosphate-buffered saline (PBS).

### Adjuvants

Squalene o/w emulsion (SE) was obtained from Polymun (Klosterneuburg, Austria) and is composed of 5% w/v squalene, 0.5% w/v sorbitane trioleate, 0.5% w/v polysorbate 80 in 10 mM sodium citrate buffer (pH 6.3). MPLA lipsomes (1.0 mg/mL) were also obtained from Polymun (Klosterneuburg, Austria). ISCOMATRIX (3,233 IU/ml) was obtained from CSL Ltd., Parkville, Victoria, Australia. GLA-LSQ was obtained from IDRI and is a composition of 0.1 mg/mL GLA in liposomes and 0.04 mg/mL QS21. All adjuvants were mixed with antigen and PBS just before administration.

### Immunizations

In a first immunization study, 24 rabbits (New Zealand White, female, four groups, 6 animals/group) were immunized under subcontract at the National Food Chain Safety Office, Directorate of Veterinary Medicinal Products (NFCSO-DVMP, Budapest, Hungary) with 20 µg of PGT145-purified ConM SOSIP.v7 protein by two intramuscular immunizations in each quadriceps at week 0, 4 and 20. The first group received a soluble ConM SOSIP.v7 trimer without adjuvant. The second and third group received the same immunogen formulated with 1:1 v/v squalene emulsion (SE) adjuvant or with 50 μL v/v MPLA liposomes (MPLA) adjuvant, respectively. The fourth group received 20 µg ConM SOSIP.v7-ferritin nanoparticles formulated in SE adjuvant. 5 mL blood samples were taken from the ear median artery at week 0, 4, 6, 16, 20, and 22. All procedures were approved by the animal ethics committee of NFCSO-DVMP.

In a second immunization study^11^, 10 rabbits (New Zealand White, female, two groups, five animals/group) were immunized with either 22 µg of ConM SOSIP.v7 trimer or ConM SOSIP.v7-ferritin nanoparticles formulated in 75 units of ISCOMATRIX adjuvant (CSL Ltd., Parkville, Victoria, Australia) by two intramuscular immunizations in each quadriceps at week 0, 4, and 20.

In a third immunization study^56^, 10 rabbits (New Zealand White, female, two groups, five animals/group) were immunized with either 30 µg of ConM SOSIP.v7 trimer or 38 µg ConM SOSIP.v7-ferritin nanoparticles formulated in GLA-LSQ adjuvant (25 µg GLA and 10 µg QS21 from IDRI) by two intramuscular immunizations in each quadriceps at week 0, 4, and 20. The second and third immunization studies were performed under subcontract at Covance (Denver, USA). The immunization procedures were carried out under the ethical guidelines and protocols approved by the Covance Institutional Animal Care and Use Committee (IACUC). All trimer and nanoparticle doses are indicated in protein content only; i.e., glycans are ignored in recording amounts. All studies were conducted under applicable laws and guidelines for animal testing.

### Enzyme-linked immunosorbent assay (ELISA)

ELISAs were performed essentially as described before^11,56^. Purified ConM SOSIP.v7 trimers (1.0 μg/mL) were diluted in TBS and immobilized on 96-well Ni-NTA ELISA plates (Qiagen) for 2 h and subsequently washed with TBS. Sera were serially diluted in 2% skimmed milk and 20% sheep serum in TBS and incubated for 2 hs. After washing with TBS, 1:3000 diluted HRP-labeled goat antirabbit IgG (111-035-144; Jackson Immunoresearch) in TBS + 2% skimmed milk was added for 1 h. Next, after washing the plates five times with TBS + 0.05% Tween-20, developing solution (1% 3,3′,5,5′-tetranethylbenzidine (Sigma-Aldrich), 0.01% H_2_O_2_, 100 mM sodium acetate, and 100 mM citric acid) was added. Colorimetric development was terminated by adding 0.8 M H_2_SO_4_. For determining anti-ferritin titers, 2.0 μg/mL of naked ferritin cages^11^ were coated overnight on half-well 96-well plates, which were then blocked using Blocker Casein (Thermo Scientific). Half-maximal binding titers (EC_50_) were calculated using GraphPad Prism 8.3.

### Neutralization assay

TZM-bl neutralization assays were performed essentially as described elsewhere^7,65^. TZM-bl reporter cell line was obtained through the NIH AIDS Research and Reference Reagents Program, Division of AIDS, NIAID, NIH (John C. Kappes, Xiaoyun Wu and Tranzyme Inc., Durham, NC, USA). One day prior to virus infection, 1.7 × 10^4^ TZM-bl cells per well were seeded in a 96-well plate in DMEM containing 10% FCS, 1 × MEM nonessential amino acids, penicillin and streptomycin (both at 100 U/mL), and incubated at 37 °C for 24 h in an atmosphere containing 5% CO_2_. To determine the neutralization activity of rabbit sera, the virus was incubated for 1 h at room temperature with heat-inactivated sera (threefold serial dilutions starting at 1:20). The mix was then added to the cells in the presence of 40 μg/mL DEAE-Dextran (Sigma) and Saquinavir, in a total volume of 200 μL. Three days later, the medium was removed and the cells were lysed in Reporter Lysis Buffer (Promega). Luciferase activity was measured using a Luciferase Assay kit (Promega) and a Glomax Luminometer according to the manufacturer’s instructions (Turner BioSystems). All infections were performed in duplicate. Uninfected cells were used to correct for background luciferase activity. All data shown in the main figures were generated at the AMC. Serum from week 22 was also tested by OSR and/or DUMC (Duke University Medical Center, Durham, NC, USA) (displayed in Supplementary Table 2). All DUMC data in Supplementary Table 2 was published before^11,56^. The NAb titers determined in the three labs correlated well (Supplementary Fig. 1b–d). The ID_50_ values were determined as the sera dilution at which infectivity was inhibited by 50%. To assess the induction of autologous NAb responses, we tested the neutralizing activity of the sera against the infectious molecular clone (IMC) carrying the autologous ConM Env^11^.

### Statistical analyses

Measurements were taken from distinct serum samples. We used nonparametric tests to analyze results, since most data were not normally distributed. Multiple groups were compared using a Kruskal–Wallis test followed by Dunn’s post-test. When two groups were compared, an unpaired two-tailed Mann–Whitney *U* test was used, unless noted otherwise. Spearman’s rank correlation coefficient was used to determine correlations. All statistical analyses were performed in GraphPad Prism 8.3.

### Reporting Summary

Further information on research design is available in the Nature Research Reporting Summary linked to this article.

## Supplementary information

Supplementary materials

REPORTING SUMMARY

## Data Availability

The data that support the findings in this study are available from the corresponding author (R.W.S.) upon reasonable request.
