# Author Correction: Interplay of diverse adjuvants and nanoparticle presentation of native-like HIV-1 envelope trimers

**DOI:** 10.1038/s41541-021-00398-1

**Published:** 2021-11-02

**Authors:** Kwinten Sliepen, Edith Schermer, Ilja Bontjer, Judith A. Burger, Réka Felfödiné Lévai, Philipp Mundsperger, Philip J. M. Brouwer, Monica Tolazzi, Attila Farsang, Dietmar Katinger, John P. Moore, Gabriella Scarlatti, Robin J. Shattock, Quentin J. Sattentau, Rogier W. Sanders

**Affiliations:** 1 Department of Medical Microbiology, Amsterdam Institute for Infection and Immunity, Amsterdam UMC, University of Amsterdam, Amsterdam, The Netherlands; 2 Control Laboratory of Veterinary Medicinal Products and Animal Facility, Directorate of Veterinary Medicinal Products, National Food Chain Safety Office, Budapest, Hungary; 3 Polymun Scientific Immunbiologische Forschung GmbH, Klosterneuburg, Austria; 4 Viral Evolution and Transmission Unit, Division of Immunology, Transplantation and Infectious Diseases, IRCCS Ospedale San Raffaele, Milan, Italy; 5 Department of Microbiology and Immunology, Weill Medical College of Cornell University, New York, NY USA; 6 Imperial College London, Department of Medicine, Division of Infectious Diseases, Section of Virology, Norfolk Place, London, W21PG UK; 7 The Sir William Dunn School of Pathology, The University of Oxford, Oxford, OX13RE UK

**Keywords:** Adjuvants, Recombinant vaccine, Protein vaccines, HIV infections

Correction to: *npj Vaccines* 10.1038/s41541-021-00364-x, published online 17 August 2021.

In this article the author name Attila Farsang was incorrectly written as Atilla Farsang. The original article has been corrected.

