# Supplementary material for: Interplay of diverse adjuvants and nanoparticle presentation of native-like HIV-1 envelope trimers
Source: NPJ Vaccines. 2021 Aug 17;6:103. doi: 10.1038/s41541-021-00364-x (PMC8371121; doi:10.1038/s41541-021-00364-x)
Supplement: Supplementary file 1 — Supplementary materials [file 41541_2021_364_MOESM1_ESM.pdf]

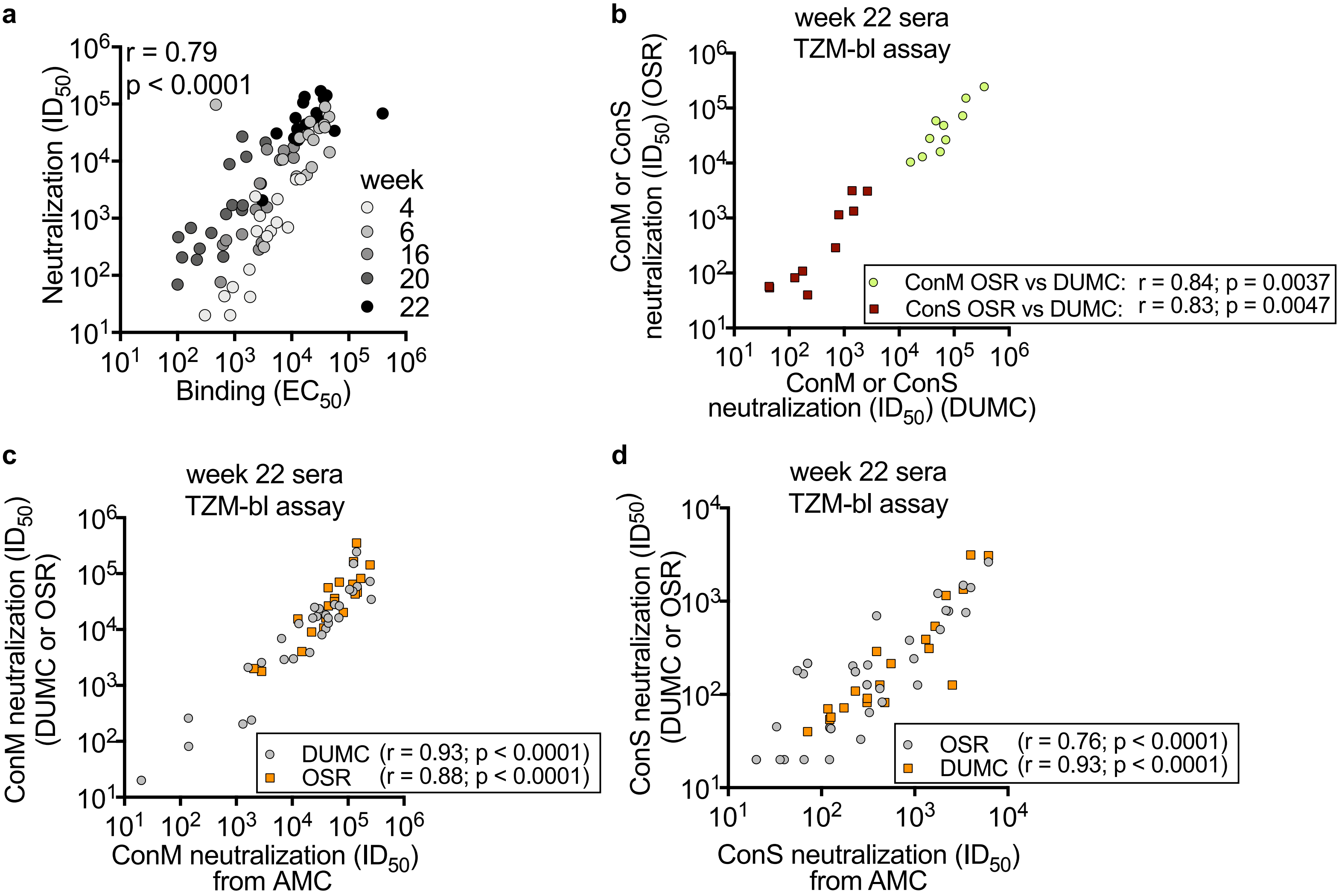

Supplementary Figure 1. Binding Ab and NAb titer correlation plots. a Linear regression analysis of the midpoint binding titers and ConM neutralization titers over all post-prime time points. **b** Correlation plot of ConM and ConS NAb titers obtained by DUMC and OSR (from SI Table 2). **c** Correlation plot of ConM NAb titers from AMC versus DUMC and OSR (from SI Table 2). **d** Correlation plot of ConS NAb titers from AMC versus DUMC and OSR (from SI Table 2). In each panel the Spearman r-values and p-values are indicated.

Supplementary Table 1

ConM SOSIP.v7 trimer midpoint binding titers (EC_{50})

Immunogen	Adjuvant	Rabbit ID	0	4	6	16	20	22 weeks
ConM trimer	Squalene emulsion	1	100	2813	13358	1655	482	12324
		2	165	1103	26171	1916	372	9958
		3	100	1500	40124	1483	152	4996
		4	100	1096	19285	4752	576	23658
		5	100	132	22242	100	100	4551
		6	100	1265	21827	1052	193	3858
	MPLA liposomes	31	100	750	18590	553	100	14287
		32	100	164	6227	691	100	2573
		33	100	120	7394	301	100	984
		34	139	197	3991	#	#	#
		35	100	100	13283	469	100	7915
		36	100	393	3138	652	154	7826
	No Adjuvant	25	100	138	848	208	100	100
		26	100	124	100	100	100	100
		27	100	100	3997	1247	100	162
		28	100	100	280	111	100	100
		29	100	213	5777	1840	178	#
		30	100	100	1339	278	100	100
	ISCOMATRIX	1839	100	3876	32882	9911	823	27142
		1840	100	4406	26438	7300	987	59287
		1841	100	1156	19752	9514	1105	45704
		1842	100	4924	24882	7615	712	13533
		1843	100	4083	20419	7649	2114	20101
	GLA- LSQ	2378	100	1468	11862	4598	1002	9602
		2379	100	184	5702	5301	625	20774
		2380	100	100	5223	3329	516	47177
		2381	100	693	10033	5484	370	9774
		2382	100	1031	16022	6593	748	40089

ConM-NP	Squalene emulsion	19	100	4325	20997	2879	713	15913
		20	100	2305	45381	3688	1360	12854
		21	100	1820	46402	2686	633	56645
		22	100	5635	38386	3719	909	391220
		23	100	2455	13729	629	103	5418
		24	100	3698	19850	714	218	11109
	ISCOMATRIX	1809	100	11905	30668	10899	1359	27980
		1810	100	8642	24132	6295	1613	17776
		1811	100	5511	37117	10850	1390	27203
		1812	100	2781	38209	7290	812	36647
		1813	100	14320	472	14059	3510	40660
	GLA-LSQ	2388	100	934	3268	569	100	3053
		2389	100	665	18458	2774	246	32331
		2390	100	302	6952	1353	120	11713
		2391	100	837	12143	2371	172	16899
		2392	100	1868	22440	3012	391	12578

#: deceased animal

Supplementary Table 2

Neutralization titers (ID50)

	week 22	Virus	MLV***	ConM			ConS			SF162	MW965.26	SHIVp3		TRO.11		25710-2.43		TV1.21		TV1.29	BG505	REJO4541.67	WITO4160.33	Ce1176_A3	AMC008	ZM197M
		Tier		1A			1B			1A	1A	2		2		2		2		2	2	2	2	2	1B	1B
		Clade		"M"			"M"			B	C	B		B		C		C		C	A	B	B	C	B	C
Immunogen	Adjuvant	Rabbit ID	AMC	AMC	OSR	DUMC	AMC	OSR	DUMC	AMC	DUMC	AMC	OSR	OSR	DUMC	OSR	DUMC	AMC	DUMC	OSR	AMC	AMC	AMC	DUMC	AMC	AMC
ConM trimer	Squalene emulsion *	1	<20	6,679	2,107		423	116		<20			<20	<20		<20		23		<20						
		2	<20	10,497	3,012		448	83		<20			<20	<20		<20		33		<20						
		3	<20	20,557	3,893		264	33		<20			<20	<20		<20		<20		<20						
		4	<20	258,754	34,782		327	64		<20			<20	<20		<20		24		<20						
		5	<20	1,310	204		65	20		<20			<20	<20		<20		24		<20						
		6	<20	6,474	6,944		64	166		<20			<20	<20		<20		27		<20						
	MPLA liposomes	31	<20	13,129	12,788		217	203		<20			<20	<20		<20				<20						
		32	<20	7,212	2,910		33	45		<20			<20	<20		<20				<20						
		33	<20	2,830	2,578		40	<20		<20			<20	<20		<20				<20						
		34	#	#	#		#	#		#			#	#		#				#						
		35	23	39,818	18,242		978	242		24			<20	<20		<20				<20						
		36	<20	27,737	17,121		313	207		<20			<20	<20		<20				<20						
	No Adjuvant	25	125	1,875	241		55	180		85			42	28		23				102						
		26	<20	20	<20		<20	<20		<20			<20	<20		<20				<20						
		27	<20	139	260		36	<20		<20			<20	<20		<20				<20						
		28	55	20	<20		122	<20		87			<20	<20		<20				<20						
		29	#	#	#		#	#		#			#	#		#				#						
		30	<20	141	81		<20	<20		<20			<20	<20		<20				<20						
	ISCOMATRIX *	1839	<20	145,975	58,493	46,649	231	175	109	29	449	<20			<20		<20		<20		<20	<20	<20	<20	<20	<20
		1840	<20	247,959	72,497	143,538	308	127	82	26	218	37			<20		<20		<20		<20	34	<20	<20	<20	<20
		1841	<20	122,086	48,667	64,681	123	44	54	<20	116	<20			<20		<20		<20		<20	20	<20	<20	<20	<20
		1842	<20	39,850	10,482	16,106	71	216	40	24	114	<20			<20		<20		<20		<20	40	<20	<20	<20	<20
		1843	<20	44,399	13,017	26,484	126	43	57	27	76	<20			<20		<20		<20		<20	<20	<20	<20	<20	<20
	GLA- LSQ **	2378	45	14,943		4,043	476		82	113	30				<20		<20							<20		
		2379	<20	11,636		1,788	174		72	<20	945				<20		<20							<20		
		2380	<20	22,379		9,042	422		126	<20	43				<20		<20							<20		
		2381	28	12,627		15,481	1,422		312	48	199				41		39							40		
		2382	<20	81,860		20,304	1,311		391	<20	1,062				<20		<20							<20		
ConM-NP	Squalene emulsion *	19	23	106,871	52,368		3,548	757		488			<20	<20		<20		127		<20						
		20	<20	23,329	16,086		1,877	496		192			<20	<20		<20		<20		<20						
		21	<20	34,131	8,033		1,771	1,211		691			<20	<20		<20		422		<20						
		22	<20	68,566	16,255		1,072	126		8,650			<20	<20		<20		169		<20						
		23	<20	30,439	23,534		880	382		178			<20	<20		<20		60		<20						
		24	<20	25,227	25,108		2,306	782		1,231			<20	<20		<20		123		<20						
	ISCOMATRIX *	1809	<20	57,783	28,178	36,017	2,163	797	1,149	52	21,599	<20			<20		<20		207		<20	<20	<20	<20	<20	<20
		1810	<20	43,914	16,014	56,137	391	696	290	61	3,732	<20			<20		<20		107		<20	<20	<20	<20	<20	<20
		1811	<20	69,643	26,597	70,273	3,321	1,485	1,344	583	9,982	62			<20		<20		158		<20	26	<20	<20	<20	<20
		1812	<20	124,964	152,493	164,177	6,199	2,639	3,090	1,437	3,486	<20			<20		<20		65		<20	<20	<20	<20	<20	<20
		1813	<20	142,221	245,476	354,473	3,993	1,399	3,141	274	29,493	<20			<20		<20		410		<20	<20	<20	<20	<20	<20
	GLA-LSQ **	2388	<20	2,056		1,999	117		70	284	2,051				<20		<20							21		
		2389	<20	168,681		81,961	2,528		126	151	1,393				<20		<20							<20		
		2390	<20	57,057		31,387	556		214	114	2,110				<20		<20							<20		
		2391	<20	133,437		43,325	1,658		539	488	2,965				<20		<20							<20		
		2392	<20	36,544		10,638	310		91	43	2,396				<20		<20							<20		

Immunizations were carried out by Covance (Denver, USA) or NFCSO-DVMP (Gödölló, Hungary).
The neutralization assays were performed at Amsterdam UMC (AMC) or Ospedale San Raffaele (OSR). Data from Duke University Medical Center (DUMC) are historical data (Brouwer et al. 2019 or Sliepen et al. 2019).
The boxes are colored according to their magnitude: white: ID50 <20; light grey: ID50 20-39; yellow: ID50 = 40-99; orange: ID50 = 100-999 ; red: ID50 = 1,000-9,999; pink: ID50 = 10,000-99,999; purple: ID50 > 100,000.

Virus/serum combinations that were not tested are colored in dark grey.
*: heterologous virus ID50 titer values obtained from Sliepen et al. 2019
**: heterologous virus ID50 titer values obtained from Brouwer et al. 2019
***: ID50 values against all other viruses are only highlighted when no significant MLV neutralization was detected (ID50 <20) or when the ID50 value is 3-fold higher than the MLV ID50 value.
#: deceased animal

Supplementary Table 3

Ferritin midpoint binding titers (EC_{50})

Immunogen	Adjuvant	Rabbit ID	w0	w4	w6	w16	w20	w22
ConM-NP	Squalene emulsion	19	50	198	1609	331	746	5640
		20	50	188	2666	371	204	2682
		21	50	329	5269	308	569	24302
		22	50	589	5321	246	221	9412
		23	50	238	1267	145	209	1969
		24	50	643	3725	390	816	10109
	ISCOMATRIX	1809	50	3738	12203	1344	1718	15382
		1810	50	2028	15302	1506	3267	30091
		1811	50	1042	8853	564	1287	11926
		1812	50	1689	4570	666	1450	3173
		1813	50	2195	17842	2355	5757	16719
	GLA-LSQ	2388	50	1946	4998	165	888	1081
		2389	50	1030	15420	592	555	4508
		2390	50	678	5539	438	732	3234
		2391	50	635	2976	1071	1139	13396
		2392	50	826	3978	427	281	3712
